# Meaning in Life and Meaningful Engagement in the Moment: Lives of Prosocial Leaders and Volunteers

**DOI:** 10.1093/geroni/igaa057.2084

**Published:** 2020-12-16

**Authors:** Jeanne Nakamura, Ajit Mann

**Affiliations:** Claremont Graduate University, Claremont, California, United States

## Abstract

Recent years have seen growing interest in older adults’ sense of meaning in life, a core dimension of eudaimonic well-being throughout adulthood that has been associated in later life with reduced morbidity and mortality. Currently, the relations between this global sense of meaning in life and the experience of meaningful engagement in the moment remain largely unexplored, particularly in later lives that are distinguished by high levels of meaning. Multilevel analysis of ESM data from prosocial leaders and volunteers indicated that feelings of meaningful engagement fluctuated in daily life for both groups, even while questionnaire data showed that global sense of meaning in life was high. Examining basic sources of fulfillment (e.g., sense of relatedness) revealed type of involvement (leadership vs. volunteerism) affected the source(s) of fulfillment that connected meaning at the global and momentary levels. Implications for theory, research, and applied work on meaning and prosocial commitment are discussed.

